# Supplementary material for: Content-rich biological network constructed by mining PubMed abstracts
Source: BMC Bioinformatics. 2004 Oct 8;5:147. doi: 10.1186/1471-2105-5-147 (PMC528731; doi:10.1186/1471-2105-5-147)
Supplement: Additional File 5 — The original Chilibot query results of the term "long-term potentiation (LTP)" and 22 other terms, limiting the latest references analyzed to the years 1990, 1995, 2000, and 2004. [file 1471-2105-5-147-S5.bz2 › chilibotAdditionalFile5/ltp2004/html/CREB_NMDA.html]

 


 **CREB** and **NMDA** 
  
Found 71 abstracts in PubMed,  **30 abstracts were retrieved and analyzed**.  


---

 Search Google  |
 PDF files only 
|  EDU domain only 

---

**Interactive relationship** (e.g. stimulation, inhibition, etc)

- Co incubation of a subthreshold dose of a group I mGluR agonist dihydroxyphenylglycine that itself did not alter basal pCREB expression augmented  **NMDA**  induced  **CREB**  phosphorylation.  Ref: 12421605 Neuroscience, 2002
- Calcium entry through synaptic  **NMDA**  receptors induced  **CREB**  activity and brain derived neurotrophic factor gene expression as strongly as did stimulation of L type calcium channels.  Ref: 11953750 Nat Neurosci, 2002
- The mGluR5 antagonist 2 methyl 6 pyridine hydrochloride blocked the DHPG augmentation of  **NMDA**  induced  **CREB**  phosphorylation, while the mGluR1 antagonist 7 cyclopropa b chromen 1a carboxylate ethyl ester did not.  Ref: 12421605 Neuroscience, 2002
- Interestingly, the protein kinase C inhibitors chelerythrine and Gö6983 also prevented DHPG from enhancing  **CREB**  phosphorylation induced by  **NMDA** .  Ref: 12421605 Neuroscience, 2002
- The  **CREB**  phosphorylation was independent of kainate AMPA receptors but partially dependent of concomitant  **NMDA**  receptor activation.  Ref: 12181423 Mol Pharmacol, 2002
- The neuronal viability and the deficiency of  **CREB**  activity, are both rescued by the pharmacological activation of the  **NMDA**  receptor.  Ref: 12405962 Eur J Neurosci, 2002
- We show that  **NMDA**  activates transcription of  **CREB**  responsive genes in hippocampal neurons, with ERK responsible for persistent  **CREB**  phosphorylation and CaM kinase IV CaMKIV responsible for phosphorylating the  **CREB**  coactivator, CBP.  Ref: 11970865 Neuron, 2002
- The dialogue between the synaptic  **NMDA**  receptors and the nucleus is also modulated by extrasynaptic  **NMDA**  receptors, which shut down activity of CRE binding protein  **CREB**  and antagonize the increase in brain derived neurotrophic factor BDNF expression induced by synaptic  **NMDA**  receptors.  Ref: 12850222 Curr Opin Neurobiol, 2003
- We propose that  **NMDA**  receptor stimulation can activate Erk1 2 and Akt signalling pathways in a PI 3 kinase dependent manner which may target  **CREB**  in the nucleus.  Ref: 11902114 J Neurochem, 2002
- These results indicate that calcium coupled group I mGluRs possess the ability to up regulate  **CREB**  phosphorylation via the intracellular calcium release induced activation of L type calcium channels and, to a lesser extent,  **NMDA**  receptors in primary striatal neurons.  Ref: 12181423 Mol Pharmacol, 2002
- Here we report that synaptic and extrasynaptic  **NMDA**  receptors have opposite effects on  **CREB**  function, gene regulation and neuron survival.  Ref: 11953750 Nat Neurosci, 2002
- Moreover, blockade of  **NMDA**  receptor NMDAR did not decrease FAK, MAPK ERK, and  **CREB**  phosphorylation although LTP induction was blunted by NMDAR antagonist.  Ref: 12764094 J Neurosci, 2003

**Parallel relationship** (e.g. studied together, co-existance, homology, etc.)

- We found that activation of  **NMDA**  receptors with  **NMDA**  rapidly and concentration dependently increased the number of neurons expressing phosphorylated  **CREB**  as revealed by immunocytochemistry.  Ref: 12421605 Neuroscience, 2002
- The new synapses are enriched in  **NMDA**  receptor and it was found that their generation involves activation of  **NMDA**  receptors, PKA and  **CREB** .  Ref: 14560699 Neurol Neurochir Pol, 2003
- With the present results, we demonstrate that  **CREB**  activation, downstream of the  **NMDA**  receptor, is a necessary step to ensure survival of these neurons.  Ref: 12405962 Eur J Neurosci, 2002
- These results indicate a facilitatory regulation of an  **NMDA**  cascade to  **CREB**  phosphorylation by concurrent glutamatergic tone on mGluR5, which is probably processed via an intracellular signaling pathway involving protein kinase C.  Ref: 12421605 Neuroscience, 2002
- Here, we present evidence that D1 receptor stimulation leads to phosphorylation of the transcription factor calcium and cyclic AMP response element binding protein  **CREB**  in the nucleus by means of  **NMDA**  receptor mediated calcium signaling.  Ref: 14622123 J Neurochem, 2003
- In contrast, calcium entry through extrasynaptic  **NMDA**  receptors, triggered by bath glutamate exposure or hypoxic ischemic conditions, activated a general and dominant  **CREB**  shut off pathway that blocked induction of BDNF expression.  Ref: 11953750 Nat Neurosci, 2002
- The facilitation of hippocampus based, long lasting synaptic plasticity, which is frequently investigated in model systems such as long term potentiation LTP and in learning paradigms such as the Morris water maze, is associated with several cellular key events calcium influx through the N methyl D aspartate  **NMDA**  receptor, generation of cyclic AMP cAMP and activation of protein kinase A PKA, phosphorylation of mitogen associated protein kinase MAPK and cAMP response element binding protein  **CREB** , and subsequent transcription of plasticity associated genes.  Ref: 12668903 Mol Neurobiol, 2003
- Phosphatidylinositol 3 kinase is a central mediator of  **NMDA**  receptor signalling to MAP kinase Erk1 2, Akt PKB and  **CREB**  in striatal neurones.  Ref: 11902114 J Neurochem, 2002
- **NMDA**  receptor dependent  **CREB**  activation in survival of cerebellar granule cells during in vivo and in vitro development.  Ref: 12405962 Eur J Neurosci, 2002
- **CREB**  activity can be regulated by synaptic N methyl d aspartate receptors NMDARs and metabotropic GABA receptors, we have studied the role of the cAMP dependent signaling pathway in the regulation of the  **NMDA**  receptor subunit 1 NMDAR1, a subunit required for functional receptor formation.  Ref: 14720206 J Neurochem, 2004
- The effect of EGFP VSNL was specific to the actions of L type calcium channels, because  **CREB**  signaling after  **NMDA**  receptor stimulation remained intact.  Ref: 12716953 J Neurosci, 2003
- Such concentrations of glutamate decrease the phosphorylation of  **CREB**  at serine 133 in an N methyl D aspartate  **NMDA**  receptor dependent manner.  Ref: 12559097 Neuroscience, 2003
- Our results suggest that both  **NMDA**  and non  **NMDA**  receptors are involved in supraspinally administered KA induced pyramidal cell death in CA3 region of hippocampus in the mouse and the p ERK and the dephosphorylation of  **CREB**  protein may play an important role in CA3 region cell death of the hippocampus induced by KA administered supraspinally.  Ref: 12521295 Mol Cells, 2002
- These results provide good circumstantial evidence for a functional link between the  **NMDA**  receptor and  **CREB**  activity in promoting neuronal survival during development.  Ref: 12405962 Eur J Neurosci, 2002
- Dopamine D1 receptors mediate  **CREB**  phosphorylation via phosphorylation of the  **NMDA**  receptor at Ser897 NR1.  Ref: 14622123 J Neurochem, 2003
